# Patterns of antimicrobial resistance genes in pathogens across One Health sectors in Ireland: an *in silico* approach

**DOI:** 10.1128/spectrum.00044-26

**Published:** 2026-06-30

**Authors:** Akhil Prakash, Eva Kilcoyne, Damien Tansey, Fiona Walsh

**Affiliations:** 1 Department of Biology, Maynooth University 8798, Maynooth, County Kildare, Ireland; 2 Kathleen Lonsdale Institute for Human Health Research, Maynooth University 8798, Maynooth, County Kildare, Ireland; Universita degli Studi dell'Insubria, Varese, Italy

**Keywords:** antimicrobial resistance, pathogens, virulence genes, One Health, *in silico*

## Abstract

As part of a rapid risk assessment, an *in silico* approach was used to detect antimicrobial resistance (AMR) in pathogenic isolates from humans, animals, and the environment. A total of 11,670 genomic data sets were retrieved from the NCBI Pathogen Detection system for Ireland, which represented 47 pathogenic species, including *Salmonella enterica*, *Escherichia coli*/*Shigella* spp., *Staphylococcus aureus*, *Klebsiella pneumoniae*, and *Enterococcus faecium*. Identifying the most critical pathogenic strains over time is essential, as these organisms significantly contribute to mortality, morbidity, and hospitalization. The analysis identified 799 antimicrobial resistance genes (ARGs), including their allelic diversity, 117 plasmid replicons, and 274 virulence factors. Several critical ARGs, particularly those conferring resistance to beta-lactams, aminoglycosides, quinolones, and colistin, were common across isolates originating from human, animal, and environmental sources, suggesting shared resistance profiles across One Health sectors. *Klebsiella pneumoniae*, *E. coli*/*Shigella* spp., *S. enterica*, and *S. aureus* were the dominant hosts of these ARGs and associated mobile genetic elements. Increasing resistance across major antibiotic classes aligned with trends reported across other European countries. This study provides a national-scale *in silico* comparison of AMR across pathogens and One Health sectors using publicly available genomic data. The findings help reinforce Ireland’s AMR surveillance by showing which resistance genes are present and how they spread across critical pathogens in humans, animals, and the environment. These findings highlight the urgent need for improved antibiotic stewardship and integrated One Health surveillance to limit the emergence and spread of AMR.

**IMPORTANCE:**

Antimicrobial resistance (AMR) is a growing threat to human, animal, and environmental health. This study used publicly available genomic data to identify antimicrobial resistance genes (ARGs) in key bacterial pathogens circulating in Ireland. By analyzing over 11,000 genomes from humans, animals, and the environment, we found that several dangerous resistance genes, including those against last-resort antibiotics, were widespread across different sources. The study highlights which bacteria and resistance genes are most critical and how they may spread between humans, animals, and the environment. These insights provide a national snapshot of AMR, supporting more effective monitoring and prevention strategies. By revealing patterns of resistance and modes of transmission, our findings underscore the importance of coordinated antibiotic stewardship and One Health approaches to slow the emergence and spread of resistant infections, protecting public health and ensuring antibiotics remain effective.

## INTRODUCTION

Antimicrobial resistance (AMR) has emerged as a critical global health threat, often described as a “silent pandemic” requiring immediate action (1). Extensive use and misuse of antibiotics in human health care, livestock management, and agriculture continue to drive the emergence and dissemination of resistant pathogens (2). Such practices exert strong selective pressures on microbial communities, enabling the proliferation of drug-resistant pathogens across clinical, animal, and environmental settings (3). Globally, drug-resistant infections are predicted to cause between 700,000 and 10 million deaths annually by 2050, and the economic costs could be as high as US$100 trillion (4). In the EU/EEA, resistant infections cause more than 35,000 deaths each year, while an estimated 1,480 (UI 1,250–1,700) deaths in Ireland in 2021 were attributed to resistant infections (5, 6).

The increasing prevalence of multidrug-resistant (MDR) pathogens in hospitals, including carbapenem-resistant *Klebsiella pneumoniae* and methicillin-resistant *Staphylococcus aureus*, underscores the urgency of coordinated AMR mitigation strategies (7). Alarmingly, carbapenem-resistant *K. pneumoniae* has also been detected in the environment (8). The above findings emphasize the interconnected aspects associated with AMR and the importance of the One Health approach, addressing the dependence between human, animal, and environmental health aspects associated with the development and spread of AMR (9–11). Clinically important pathogens, including *Escherichia coli*, *Enterococcus faecalis*, *K. pneumoniae*, and *Salmonella enterica*, can easily acquire and disseminate resistance mechanisms, further threatening effective treatment options (12, 13).

The environment has a significant role as a reservoir and vector of AMR. Discharges from hospitals, municipal sewage systems, livestock operations, aquaculture farms, landfills, and agricultural runoff release antibiotic residues and resistant bacteria into surrounding ecosystems (14). As a result, environmental microbes accumulate antimicrobial resistance genes (ARGs) and can be detected in wildlife, including birds, without any direct exposure to antibiotics. They can disseminate resistant bacteria over large distances, including remote locations (15, 16). Studies from Ireland have detected AMR in public and private water supplies, and although overall resistance levels remain low, their presence in water supplies remains concerning (17, 18). Wastewater treatment plants (WWTPs) also represent important hotspots for AMR dissemination. Studies across Europe, including Ireland, have reported that WWTP effluents harbor resistant bacteria with patterns similar to those found in clinical settings (19–21). These environments facilitate horizontal gene transfer (HGT) via mobile genetic elements, such as plasmids, integrons, and prophages, promoting the exchange and persistence of ARGs among microbial populations (22, 23).

Ireland, like many European countries, has experienced a steady rise in AMR over the past decade (18). The reports from the Health Protection Surveillance Centre (HPSC) and the European Centre for Disease Prevention and Control highlight increasing resistance in *E. coli*, *K. pneumoniae*, and *S. aureus*, including strains resistant to carbapenems, third-generation cephalosporins, and methicillin during the period (24). Despite ongoing surveillance activities, comprehensive nationwide genomic insights remain limited from Ireland. Antibiotic stewardship programs are well established in Irish hospitals (25). However, antibiotic consumption increased by 16% from 2009 to 2019, and recent data indicate that consumption has stabilized at 76.5–76.8 defined daily doses per 100 bed days used between 2019 and 2023 (26). This suggests that stewardship efforts are well established, but resistance levels in major nosocomial pathogens have not reduced.

A systematic nationwide genomic AMR surveillance is hindered by logistical and financial barriers associated with generating new data sets. To address this gap, the current study uses publicly available whole-genome sequencing (WGS) data sets from the NCBI Pathogen Detection database as a quick and affordable way to evaluate the distribution and prevalence of virulence factors, plasmids, and ARGs in human, animal, and environmental isolates in Ireland. This study uses an *in silico* genomic framework to provide important baseline data to help policymakers, environmental managers, and healthcare professionals develop focused strategies to reduce AMR and manage future outbreaks.

## MATERIALS AND METHODS

### Data collection

WGS data were obtained from the NCBI Pathogen Detection system. This system combines bacterial genome sequences, identifying incorporated AMR and virulence genes. It enables the monitoring of the spread of resistance determinants within pathogens. We queried the database to obtain information on AMR and virulence genotype, targeting 47 pathogenic species in Ireland. The system held 11,670 records of pathogenic bacterial strains, as of June 2025. Searches were conducted using filter options to retrieve records collected between 2000 and June 2025. The filtered data set was exported as a spreadsheet (Microsoft Excel) (see File S2 at https://doi.org/10.5281/zenodo.20588678). For each entry, the following metadata were compiled: organism group, serovar, isolate, creation date, location, isolation source, virulence genotypes, and AMR genotypes.

### Analysis of antimicrobial resistance genes

The identified ARGs were categorized into their antibiotic classes using the Comprehensive Antibiotic Resistance Database (https://card.mcmaster.ca/home) and the reference genes from the NCBI Bacterial Antibiotic Resistance Reference Gene Database (https://www.ncbi.nlm.nih.gov/pathogens/refgene/). Species-wise abundance of ARGs and virulence factors was imported into RStudio for subsequent analysis. To determine plasmid-associated sequences, we used the Public Health Agency of Canada’s Staramr tool (https://github.com/phac-nml/starAMR). This pipeline integrates various databases and algorithms, including BLAST, ResFinder, PointFinder, and PlasmidFinder, enabling detection of plasmid-mediated ARGs and point mutations. For this analysis, we used the available contig assemblies downloaded from NCBI.

### Data analysis and visualization

Pathogen-wise abundance of ARGs was from the NCBI Pathogen Database and used for further analysis. The data were exported into R statistical software (v4.1.2) and converted into log values. Different packages, such as ggplot2, pheatmap, and ComplexHeatmap, were used for visualization, and dplyr and reshape2 were used for data manipulation. A principal component analysis (PCA) plot was generated using the tidyr package and Principal Coordinates Analysis (PCoA) using vegan package. A network plot was created using the tidyverse, igraph, ggraph, and tidygraph packages.

## RESULTS

### Abundance of pathogens

**Fig 1:**
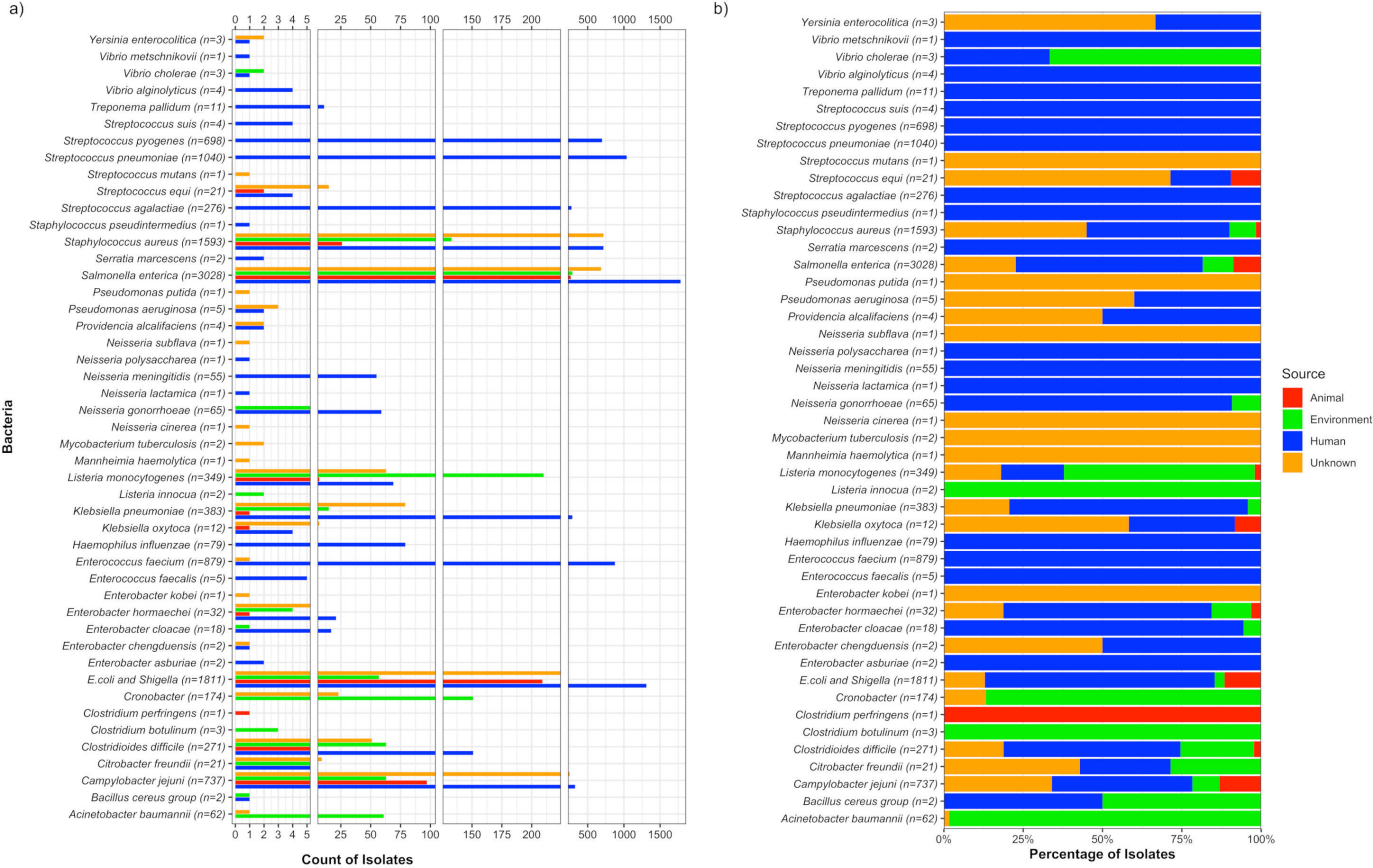
Distribution of bacterial isolates and their associated source categories. Absolute count of isolates across bacterial taxa grouped by source category (animal, environment, human, and unknown) (**a**). Relative percentage distribution of isolates according to source category for each bacterial species (**b**).

Genomic data from 47 pathogenic bacterial species were retrieved from the database. In all samples, the most abundant pathogens were *S. enterica* (*n* = 3,028, 26%), *E. coli*/*Shigella* spp. (*n* = 1811, 15.6%), and *S. aureus* (*n* = 1593, 13.8%) (Fig. 1a). Isolates were predominantly obtained from human sources (*n* = 7,822), followed by those of unknown origin (*n* = 2,160), environmental sources (*n* = 1,069), and animal sources (*n* = 619). Among pathogens of human origin, *S. enterica* (25.4%) was the most prevalent, followed by *S. aureus* (14.4%), *E. coli*/*Shigella* spp. (12.6%), *S. pneumoniae* (11.6%), and *Enterococcus faecium* (9.8%). In animal-derived isolates, *E. coli/Shigella* spp. (36%) predominated, followed by *S. enterica* (26.1%). Environmental samples were dominated by *S. enterica* (31.3%) and *S. aureus* (15.3%). The normalized percentage of isolates from each source is shown in Fig. 1b.

### Multidrug resistance and distribution of resistance-related genes

**Fig 2:**
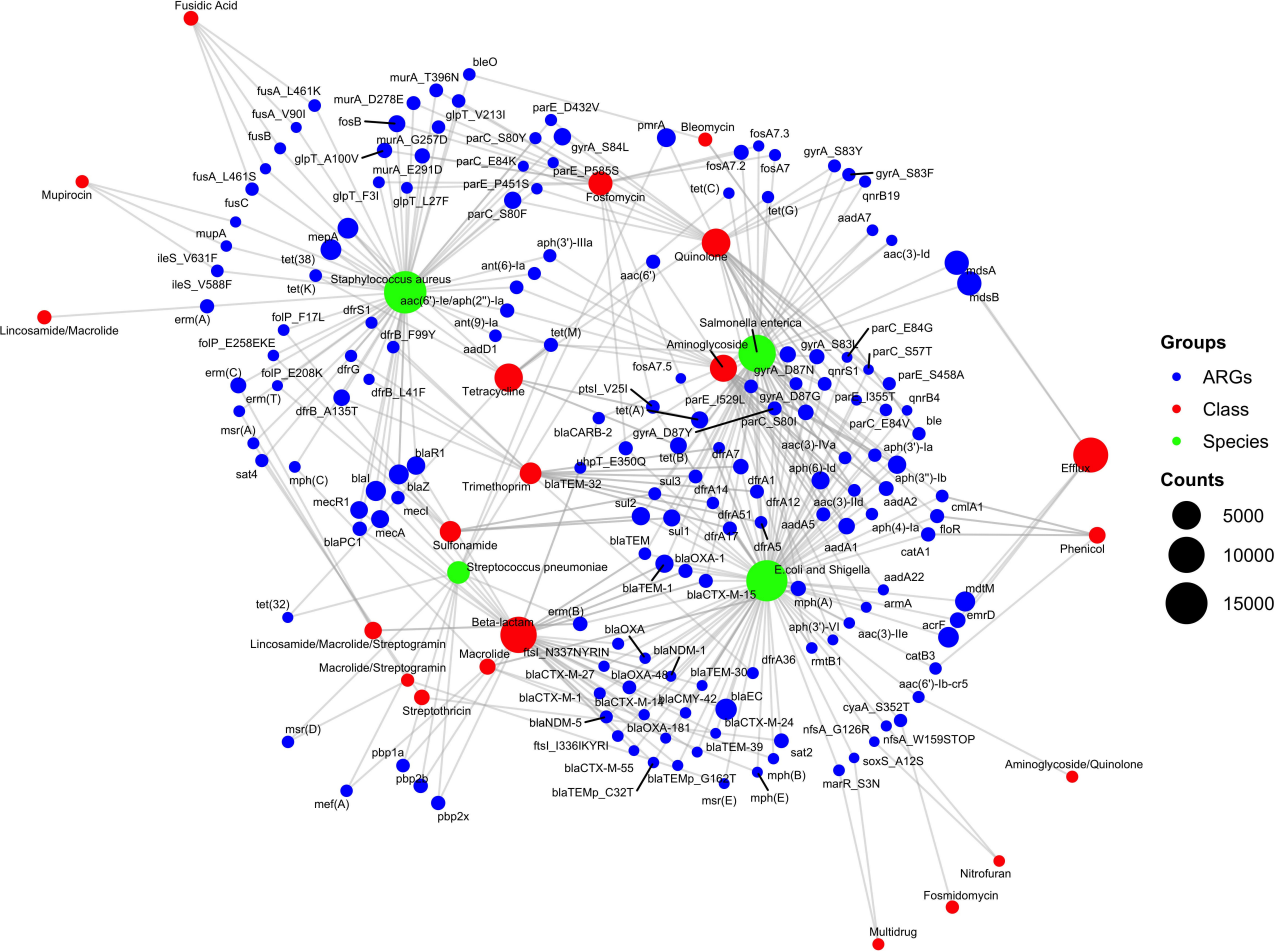
Network plot illustrating 100 major resistance-related genes associated with major pathogenic species such as *Salmonella enterica*, *Escherichia coli*/*Shigella* spp., *Staphylococcus aureus*, and *Streptococcus pneumoniae*. In the plot, blue nodes represent ARGs; red nodes indicate antibiotic classes; and green nodes denote pathogenic species.

In this study, a total of 799 resistance-related genes, including their allelic diversity, were detected across all isolates. A total of 10,896 isolates were found to carry resistant genes. The majority originated from human sources (*n* = 7,110, 65.2%), followed by isolates of unknown origin (*n* = 2,113, 19.3%), environmental sources (*n* = 1,069, 9.7%), and animal sources (*n* = 604, 5.5%). Across the four sources, 117 resistance-related genes were identified as common. Source-specific analysis revealed that the human-associated data set contained the highest number of unique resistance genes (*n* = 280, 35%), followed by the unknown origin (*n* = 81, 10.1%), the environmental source (*n* = 26, 3.3%), and the animal source (*n* = 10, 1.3%). The highest numbers of genes were observed in *K. pneumoniae* (*n* = 197) and *E. coli*/S*higella* spp. (*n* = 183), followed by *S. enterica* (*n* = 159) and *S. aureus* (*n* = 112). The most abundant resistance-related genes were *mdsB* and *mdsA*. These are associated with efflux mechanisms, followed by *blaEC*, responsible for beta-lactam resistance, and *tet(38*), which confers resistance to tetracyclines. The network plot shows the abundance of ARGs in the major pathogenic isolates like *S. enterica*, *E. coli*/S*higella* spp., *S. aureus*, and *S. pneumoniae* (Fig. 2). ARG genotypes from each isolate are listed in Table 1 and Table S1 at https://doi.org/10.5281/zenodo.20588678.

**TABLE 1:** Top 20 AMR genes identified in different pathogenic isolates from Ireland

Antimicrobial resistance gene	Source organism	Antibiotic class	ARG prevalence per genome (%)
*mdsB*	*Salmonella enterica*	Efflux	95.14
*mdsA*	*Salmonella enterica*	Efflux	95.04
*blaEC*	*E. coli* and *Shigella* spp.	Beta-lactam	99.55
*tet(38)*	*Staphylococcus aureus*	Tetracycline	100
*mepA*	*Staphylococcus aureus*	Tetracycline	99.93
*acrF*	*E. coli* and *Shigella* spp.	Efflux	86.52
*blaI*	*Staphylococcus aureus*	Beta-lactam	88.82
*mdtM*	*E. coli* and *Shigella* spp.	Efflux	77.30
*blaZ*	*Staphylococcus aureus*	Beta-lactam	83.80
*pmrA*	*Streptococcus pneumoniae*	Quinolone	100
*blaR1*	*Staphylococcus aureus*	Beta-lactam	60.01
*tet(M)*	*Enterococcus faecium*	Tetracycline	100
*msr(C)*	*Enterococcus faecium*	Macrolide/streptogramin	100
*aac(6')-I*	*Enterococcus faecium*	Aminoglycoside	99.88
*pbp5_E629V*	*Enterococcus faecium*	Beta-lactam	96.81
*pbp5_M485A*	*Enterococcus faecium*	Beta-lactam	96.70
*mecA*	*Staphylococcus aureus*	Beta-lactam	52.47
*parC_S80I*	*Enterococcus faecium*	Quinolone	93.28
*mecR1*	*Staphylococcus aureus*	Beta-lactam	48.90
*vanS-A*	*Enterococcus faecium*	Glycopeptide	88.50

**Fig 3:**
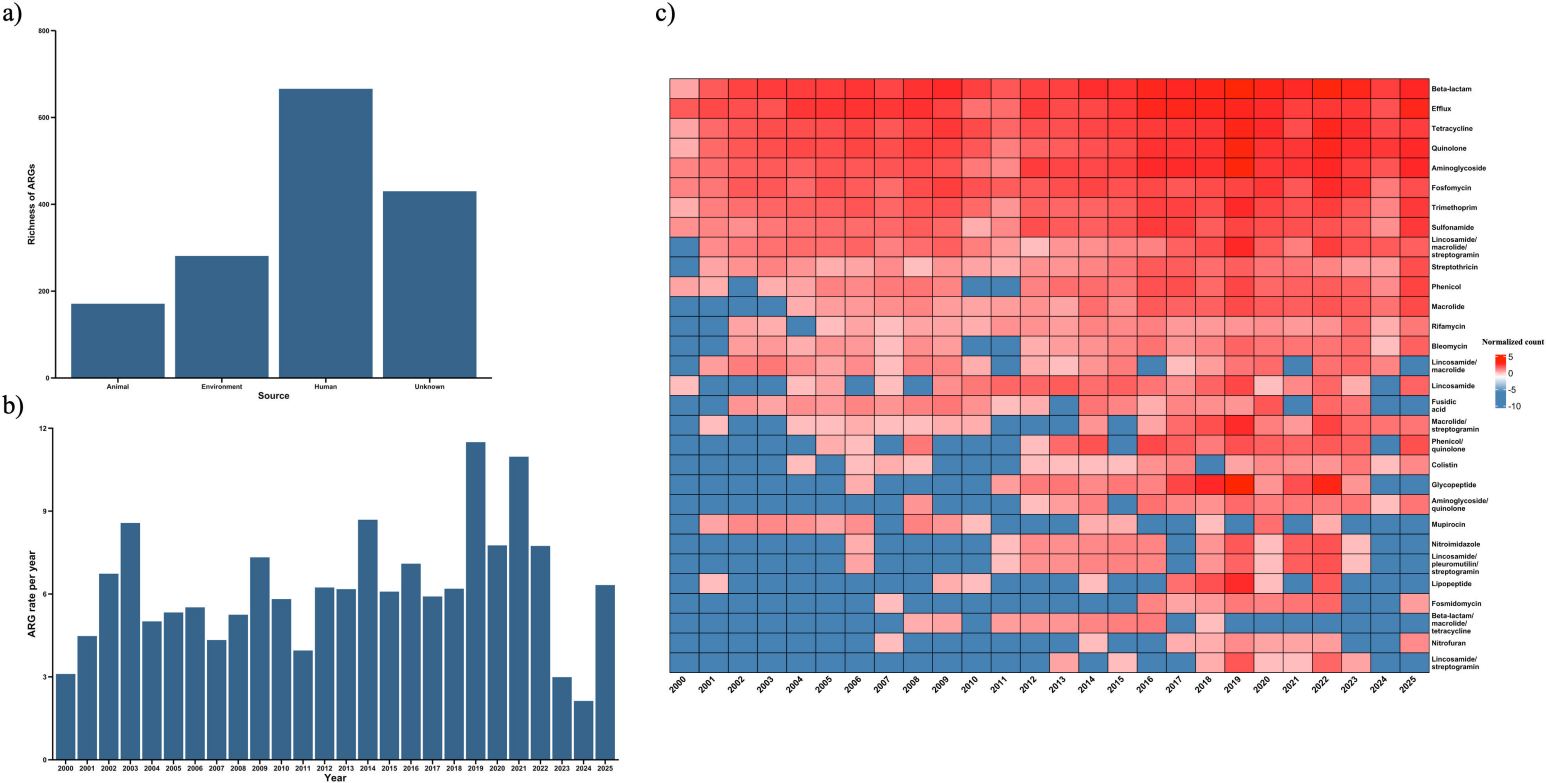
Trends in isolate genome counts , richness of ARGs across different sources (**a**), and ARG rates per year from 2000 to 2025 (**b**) . The heatmap shows the normalized abundance of different antibiotic classes across years, where red indicates higher values and light blue indicates lower values (**c**).

The spatiotemporal distribution of ARGs from 2000 to 2025 showed substantial variation across sources and over time. Human-associated samples contained the highest diversity of ARGs, with more than 600 detected genes, followed by environmental and unknown sources, whereas animal-associated sources showed comparatively lower diversity (Fig. 3a). Temporal trends indicated fluctuations in ARG reporting, with notable peaks observed in 2019, and 2021, possibly associated with increased genomic surveillance efforts during COVID-19 period or the emergence of clinically important resistance determinants (Fig. 3b). The heatmap analysis further demonstrated the persistent dominance of major ARG classes such as beta-lactam, aminoglycosides, tetracycline and quinolone resistance genes throughout the study period, while glycopeptide and nitroimidazole resistance genes became more prominent after 2011 (Fig. 3c). Overall, these findings indicate an expanding resistome within the Irish surveillance dataset, likely influenced by anthropogenic activities and ongoing dissemination of resistance mechanisms and the continued spread of resistance mechanisms across multiple ecological niches.

### Beta-lactam resistant genes

**Fig 4:**
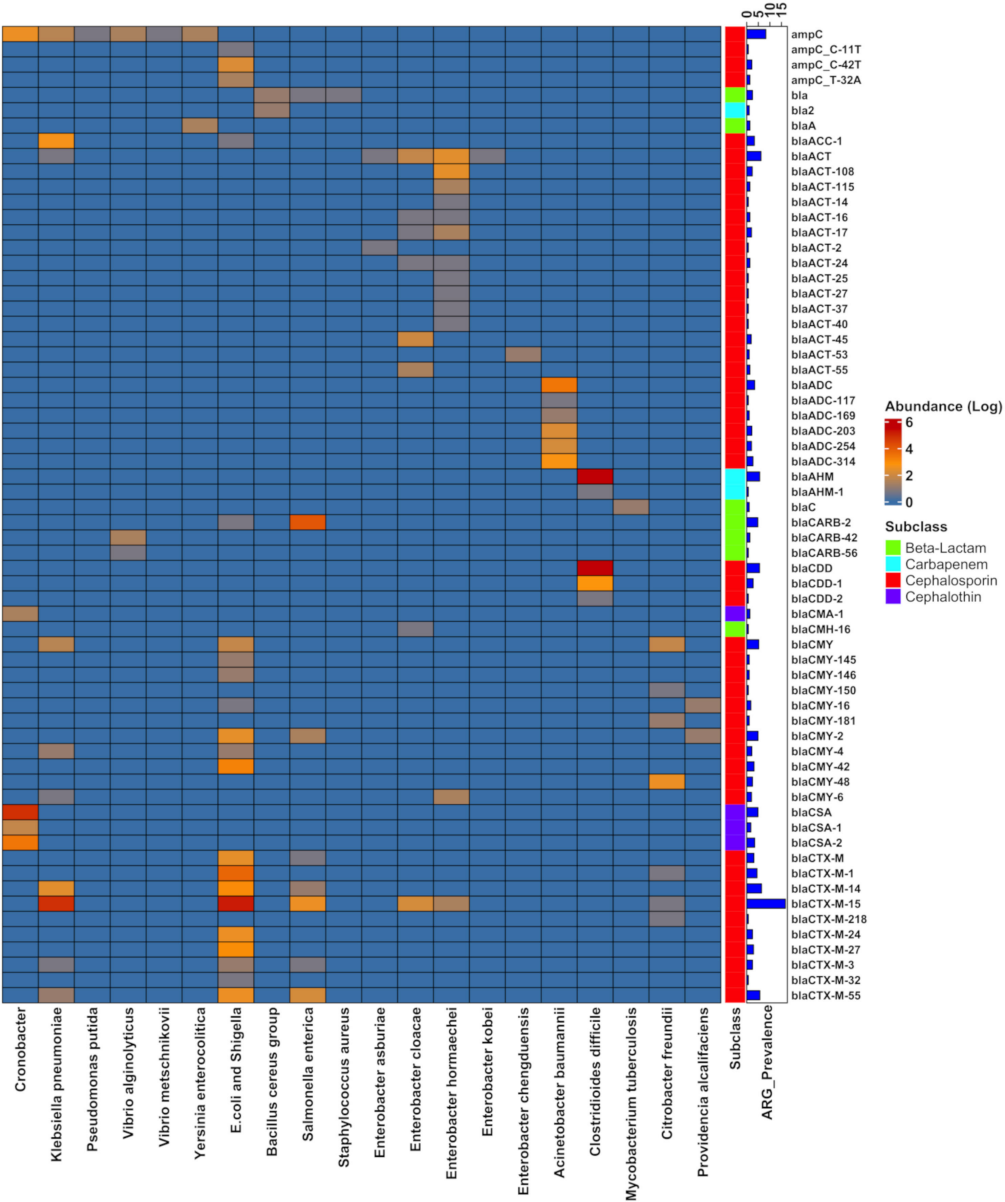
Complex heatmap showing the distribution and abundance of beta-lactam resistance genes across different bacterial taxa. Rows represent ARGs and columns represent bacterial organisms. Heatmap colors indicate log-transformed abundance values, ranging from low (blue) to high abundance (red). The right-side annotation indicates the subclass of beta-lactam antibiotic and the prevalence of beta-lactam resistance genes in the bar plot.

The analysis revealed 294 beta-lactam resistance-related variable genes in additional pathogens spanning 32 species. The majority were found in *K. pneumoniae* (*n* = 81) and *E. coli*/S*higella* spp. (*n* = 61), followed by *S. enterica* (*n* = 35), *Campylobacter jejuni* (*n* = 31), *Neisseria gonorrhoeae* (*n* = 27), *Enterobacter hormaechei* (*n* = 26), *Klebsiella oxytoca* (*n* = 25), *Enterobacter cloacae* (*n* = 20), *Citrobacter freundii* (*n* = 19), *Acinetobacter baumannii* (*n* = 18), *Haemophilus influenzae* (*n* = 17), *Clostridioides difficile* (*n* = 11), and *S. aureus* (*n* = 10) (Fig. 4; Table S1 at https://doi.org/10.5281/zenodo.20588678). The most detected beta-lactam resistance genes were *blaEC*, encoding for penicillin and cephalosporin resistance in *E. coli*/*Shigella* spp., followed by *blaZ*, responsible for penicillin resistance, as well as regulatory genes such as *blaI* and *blaR1*, mainly abundant in *S. aureus*. In *E. faecium*, the point mutation *pbp5_E629V* was the dominant mechanism responsible for resistance to penicillin and other beta-lactams.

**Fig 5:**
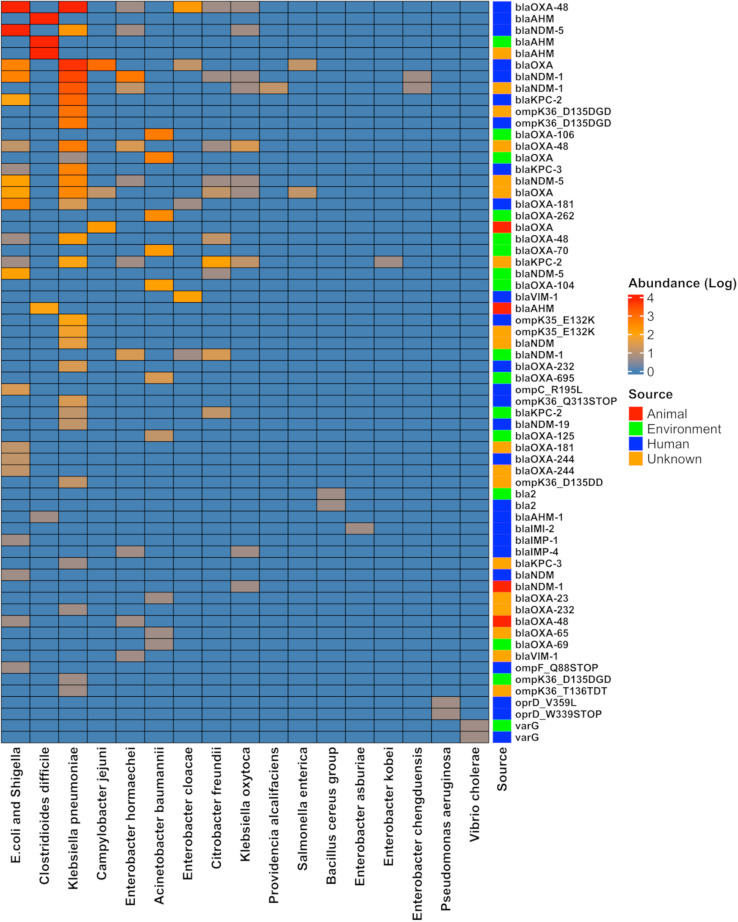
Heatmap showing the distribution and abundance of major carbapenem resistance genes across different bacterial taxa. Rows represent ARGs and columns represent bacterial organisms. Heatmap colors indicate log-transformed abundance values, ranging from low (blue) to high abundance (red). The right-side annotation indicates the source category of each ARG (animal, environment, human, and unknown).

Among beta-lactam resistance types, carbapenem resistance is particularly concerning. Thirty-seven carbapenem resistance-related genes were found, with *K. pneumoniae* (*n* = 19) showing the highest number, followed by *E. coli*/S*higella* spp. (*n* = 15). The most detected were *bla_AHM_*, which confers resistance to carbapenem, and *bla_OXA_*, which confers penicillin, cephalosporin, or carbapenem resistance, depending on the mutant version of the *bla*_OXA_ gene. In the case of the clinically important gene *bla_NDM_* (metallo-beta-lactamase), *K. pneumoniae* had the highest number, followed by *E. coli*/S*higella* spp. Five distinct *bla_NDM_* variants were identified in the data set. Bacteria carrying *bla_NDM_* genes encode enzymes that confer resistance to a broad range of beta-lactam antibiotics, including carbapenems, which are considered last-resort treatments. We also detected *bla_OXA-48_*, *bla_KPC_*, *bla_VIM_*, and *bla_IMP_* mainly from *K. pneumoniae* and *E. coli*/S*higella* spp. (Fig. 5 and Table 2). The detection of carbapenem-associated genes such as *bla_OXA-48_* is particularly concerning because carbapenems are often considered last-line therapies for MDR gram-negative infections. The widespread occurrence of ESBL-associated genes may compromise empirical treatment efficacy and increase reliance on reserve antibiotics.

**TABLE 2:** Carbapenemase gene counts normalized per pathogenic isolate from Ireland

Organism	Gene	Gene count	Species count	Normalized value
*Acinetobacter baumannii*	*blaOXA*	14	62	0.2258
	*blaOXA-104*	7	62	0.1129
	*blaOXA-106*	15	62	0.2419
	*blaOXA-125*	2	62	0.0323
	*blaOXA-23*	1	62	0.0161
	*blaOXA-262*	11	62	0.1774
	*blaOXA-65*	1	62	0.0161
	*blaOXA-69*	1	62	0.0161
	*blaOXA-695*	3	62	0.0484
	*blaOXA-70*	8	62	0.129
*Bacillus cereus* group	*bla2*	2	2	1
*Campylobacter jejuni*	*blaOXA*	30	737	0.0407
*Citrobacter freundii*	*blaKPC-2*	9	21	0.4286
	*blaNDM-1*	4	21	0.1905
	*blaNDM-5*	2	21	0.0952
	*blaOXA*	2	21	0.0952
	*blaOXA-48*	4	21	0.1905
*Clostridioides difficile*	*blaAHM*	170	271	0.6273
	*blaAHM-1*	1	271	0.0037
*E. coli* and *Shigella*	*blaIMP-1*	1	1,811	0.0006
	*blaKPC-2*	7	1,811	0.0039
	*blaKPC-3*	1	1,811	0.0006
	*blaNDM*	2	1,811	0.0011
	*blaNDM-1*	13	1,811	0.0072
	*blaNDM-5*	98	1,811	0.0541
	*blaOXA*	21	1,811	0.0116
	*blaOXA-181*	13	1,811	0.0072
	*blaOXA-244*	4	1,811	0.0022
	*blaOXA-48*	164	1,811	0.0906
	*ompC_R195L*	3	1,811	0.0017
	*ompF_Q88STOP*	1	1,811	0.0006
*Enterobacter asburiae*	*blaIMI-2*	1	2	0.5
*Enterobacter chengduensis*	*blaNDM-1*	2	2	1
*Enterobacter cloacae*	*blaNDM-1*	1	18	0.0556
	*blaOXA*	2	18	0.1111
	*blaOXA-181*	1	18	0.0556
	*blaOXA-48*	8	18	0.4444
	*blaVIM-1*	7	18	0.3889
*Enterobacter hormaechei*	*blaIMP-4*	1	32	0.0313
	*blaKPC-2*	1	32	0.0313
	*blaNDM-1*	21	32	0.6563
	*blaNDM-5*	2	32	0.0625
	*blaOXA-48*	5	32	0.1563
	*blaVIM-1*	1	32	0.0313
*Enterobacter kobei*	*blaKPC-2*	1	1	1
*Klebsiella oxytoca*	*blaIMP-4*	1	12	0.0833
	*blaKPC-2*	2	12	0.1667
	*blaNDM-1*	3	12	0.25
	*blaNDM-5*	2	12	0.1667
	*blaOXA*	1	12	0.0833
	*blaOXA-48*	4	12	0.3333
*Klebsiella pneumoniae*	*blaKPC-2*	30	383	0.0783
	*blaKPC-3*	14	383	0.0366
	*blaNDM*	2	383	0.0052
	*blaNDM-1*	48	383	0.1253
	*blaNDM-19*	2	383	0.0052
	*blaNDM-5*	11	383	0.0287
	*blaNDM-60*	1	383	0.0026
	*blaOXA*	46	383	0.1201
	*blaOXA-181*	3	383	0.0078
	*blaOXA-232*	4	383	0.0104
	*blaOXA-48*	154	383	0.4021
	*ompK35_E132K*	10	383	0.0261
	*ompK36_D135DD*	1	383	0.0026
	*ompK36_D135DGD*	27	383	0.0705
	*ompK36_Q313STOP*	3	383	0.0078
	*ompK36_T136TDT*	1	383	0.0026
*Providencia alcalifaciens*	*blaNDM-1*	2	4	0.5
*Pseudomonas aeruginosa*	*oprD_W339STOP*	1	5	0.2
*Salmonella enterica*	*blaOXA*	4	3,028	0.0013
*Vibrio cholerae*	*varG*	2	3	0.6667

### Aminoglycoside resistance genes

**Fig 6:**
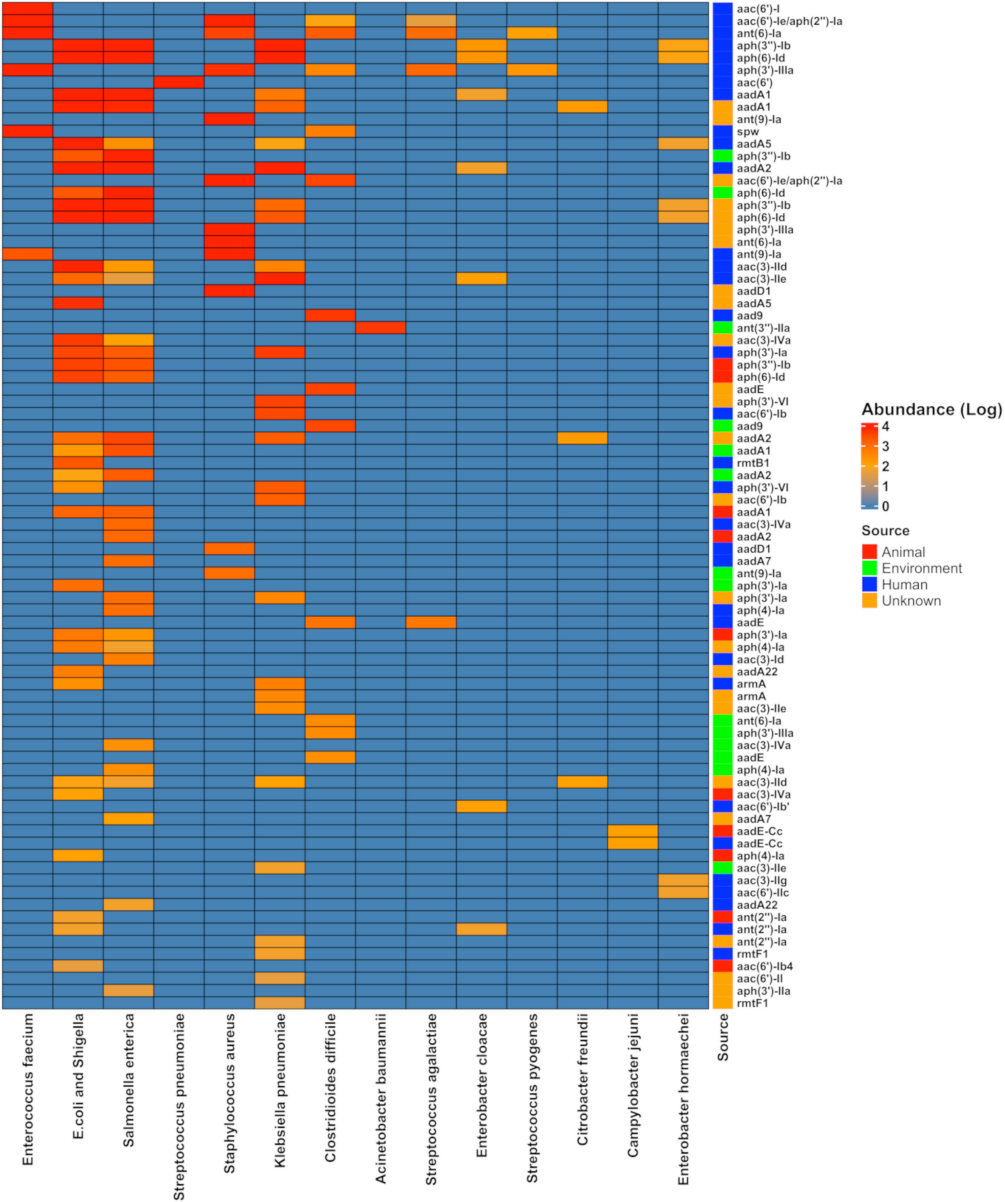
Heatmap showing the distribution and abundance of major aminoglycoside resistance genes across different bacterial taxa. Heatmap colors indicate log-transformed abundance values, ranging from low (blue) to high abundance (red). The right-side annotation indicates the source category of each ARG (animal, environment, human, and unknown).

The aminoglycoside resistance genes inactivate the class of drugs by modifying specific amino or hydroxyl groups on the drugs and include N-acetyltransferases, O-nucleotidyltransferases, and O-phosphotransferases. From the isolates, a total of 72 aminoglycoside resistance-associated genes were identified from 26 species. Most ARGs were found in *S. enterica* (*n* = 40), *K. pneumoniae* (*n* = 37), and *E. coli*/S*higella* spp.(*n* = 36) (Fig. 6). The most abundant ARGs were *aac(6*′*)-I*, *aac(6*′*)-Ie/aph(2*″*)-Ia*, and *aac(2*′*)-I(A267)*, which confer resistance to amikacin, gentamicin, kanamycin, and aminoglycosides, respectively.

### Colistin resistance genes

**Fig 7:**
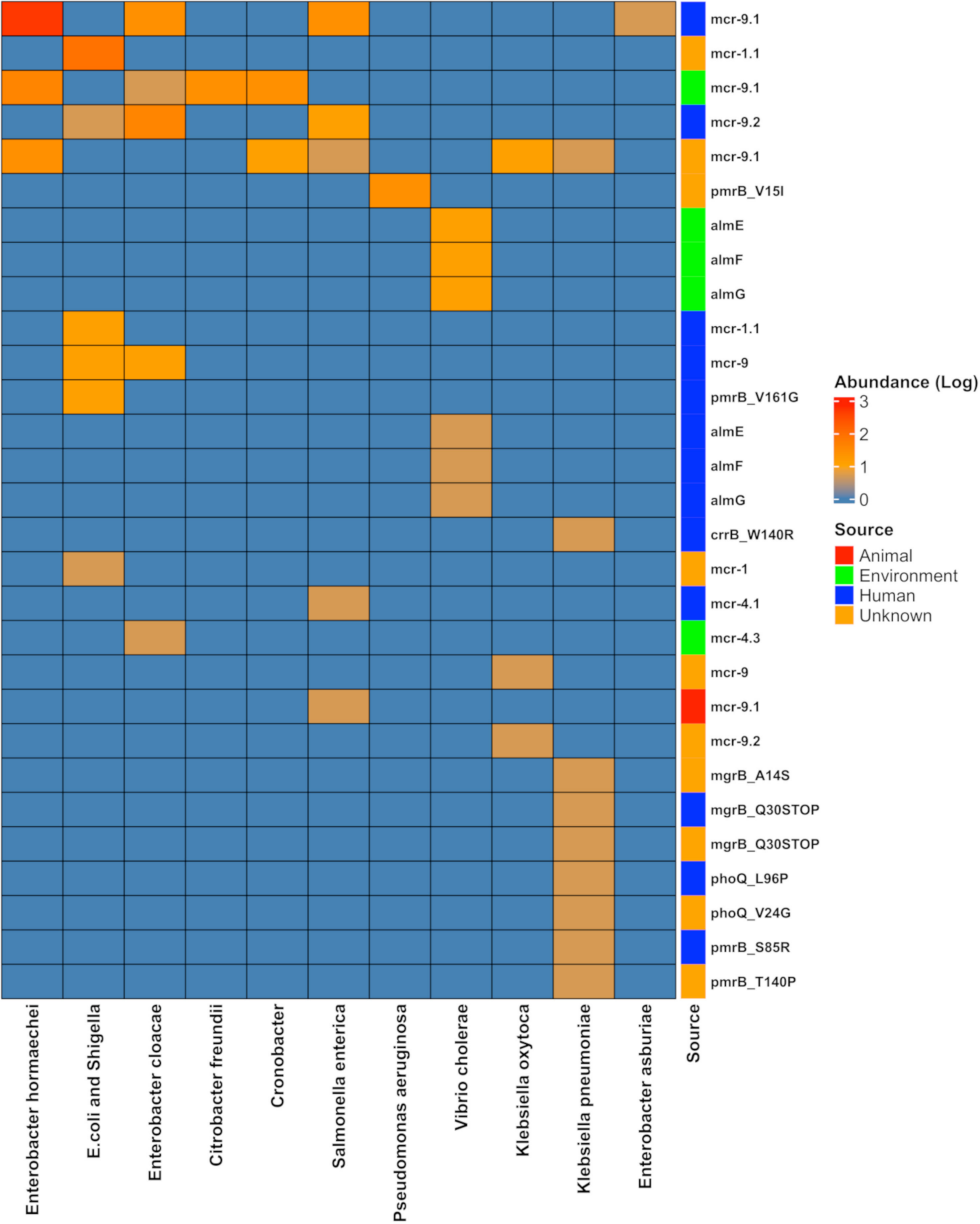
Heatmap showing the distribution and abundance of major colistin resistance genes across different bacterial taxa. Heatmap colors indicate log-transformed abundance values, ranging from low (blue) to high abundance (red). The right-side annotation indicates the source category of each ARG (animal, environment, human, and unknown).

We further carried out a detailed study on the presence of colistin resistance genes in the isolates. A high number of ARGs were detected in *K. pneumoniae* and *E. coli*/*Shigella* spp., and *E. hormaechei* exhibited a high abundance of *mcr-9.1* (Fig. 7). Colistin is a last-resort antibiotic used to treat infections caused by multidrug-resistant gram-negative bacteria. However, the rise of colistin resistance, especially due to plasmid *mcr* genes, has proven to be a major public health concern. A total of 20 colistin ARGs/mutations conferring resistance to colistin were found in 11 different species from Ireland.

**Fig 8:**
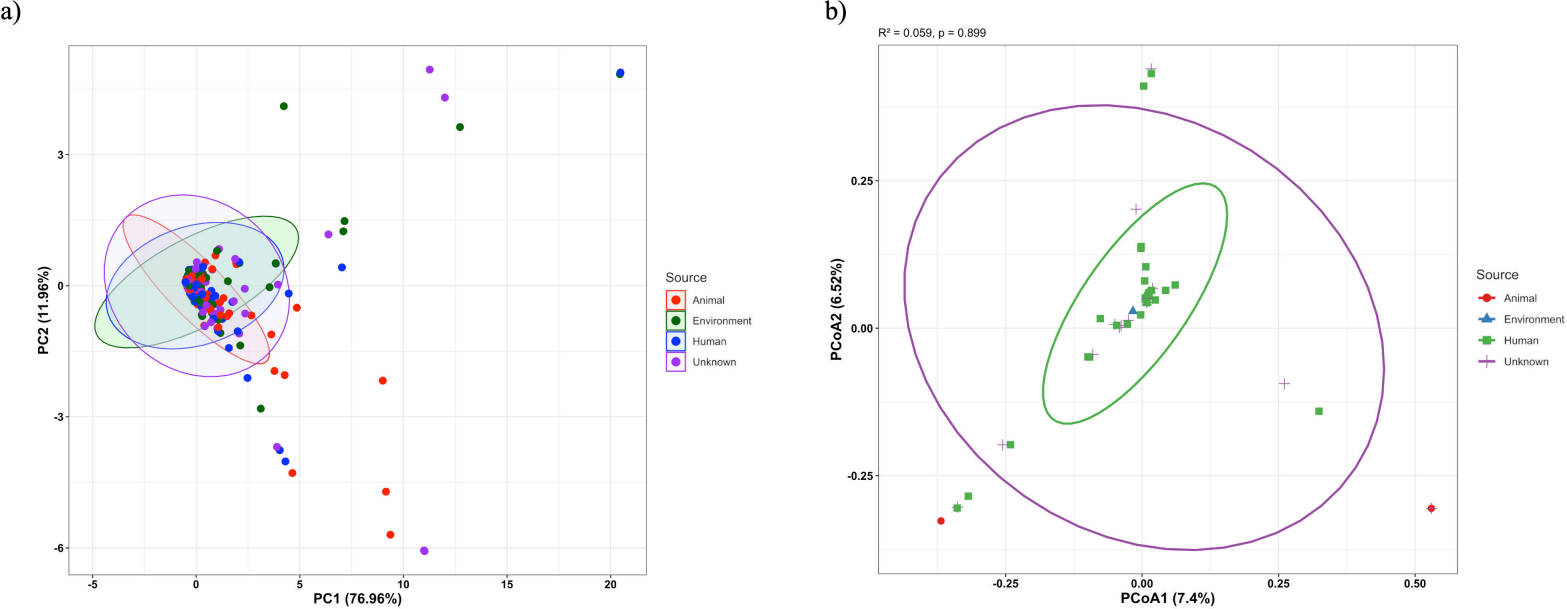
Principal component analysis (PCA) of ARG profiles in pathogenic isolates from diverse sources, illustrating clustering patterns based on similarities in ARG composition (**a**). Principal coordinates analysis (PCoA) of ARG profiles across different sources, showing the overall variation and separation of samples according to their resistance gene distributions (**b**).

The abundance of ARGs from each source was analyzed using PCA, with each point representing a single gene. The analysis shows that most ARG profiles from different sources cluster closely together, suggesting shared resistance profiles across sectors, potentially reflecting common resistance determinants (Fig. 8a). Consistent with this observation, PCoA showed no clear separation among source groups (Fig. 8b). PERMANOVA analysis revealed no significant differences in ARG composition across sources (*R*² = 0.059, *P* = 0.899). The source variable explained only a minimal proportion of the overall variation, indicating that it had a limited influence on the observed ARG profiles (Fig. 8b). This highlights the possibility that resistance genes can move between sectors and underscores why a unified One Health approach to monitoring AMR is so important.

### Plasmid replicons

**Fig 9:**
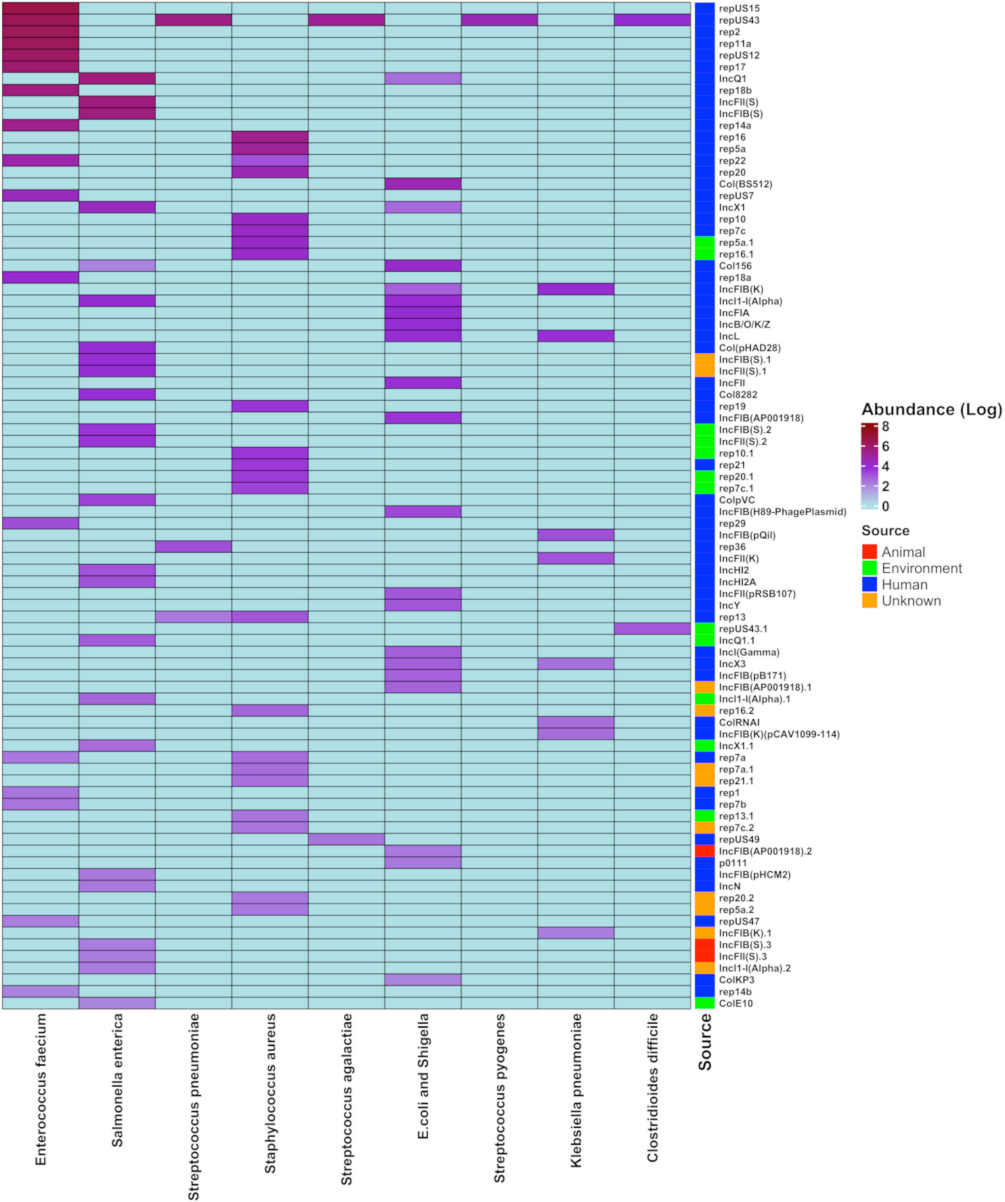
Heatmap showing the log-transformed abundance of plasmid replicons across major bacterial pathogens. Rows represent individual plasmid replicons, and columns represent bacterial species. Color intensity indicates relative gene abundance on a logarithmic scale, with darker purple denoting higher abundance and light blue indicating lower abundance. Side annotations show the source category associated with each gene profile, including animal, environmental, human, and unknown origins.

Genomes were screened for plasmid replicons, resulting in the identification of 117 replicons. Only nine plasmid replicons were shared across all four sources. The highest number of plasmid replicons was identified in isolates from the human source. Additionally, two unique replicons were detected in both the animal and unknown sources. Most of the plasmids were associated with major pathogens. *E. coli*/*Shigella* spp. exhibited a high number, accounting for 22.4% of whole plasmid replicons detected, followed by *S. enterica* (15.9%), *K. pneumoniae* (14.8%), and *S. aureus* (14.8%). The most observed plasmid replicons were *repUS43* (9.6%), *repUS15* (6.4%), and *rep5a* (4.7%) from the samples. Plasmids are usually conjugative, facilitating HGT among bacterial populations and contributing significantly to the spread of AMR. In *E. coli*/S*higella* spp., the most observed plasmids were *IncFIB(AP001918)* and *IncB/O/K/Z*, which are mainly associated with multidrug resistance and HGT. The heatmap illustrates the plasmid replicon present in each pathogenic species (Fig. 9). Both *S. aureus* and *Enterococcus* isolates harbor diverse plasmid replicon (*rep*) types, with certain plasmids highly abundant (e.g., *rep5a* in *S. aureus* and *repUS15* in *E. faecium*). All detected plasmids belong to *rep* families, reflecting species-specific replication, and likely act as key vectors for the dissemination of ARGs and other mobile genetic elements in these gram-positive pathogens.

### Virulent factors

**Fig 10:**
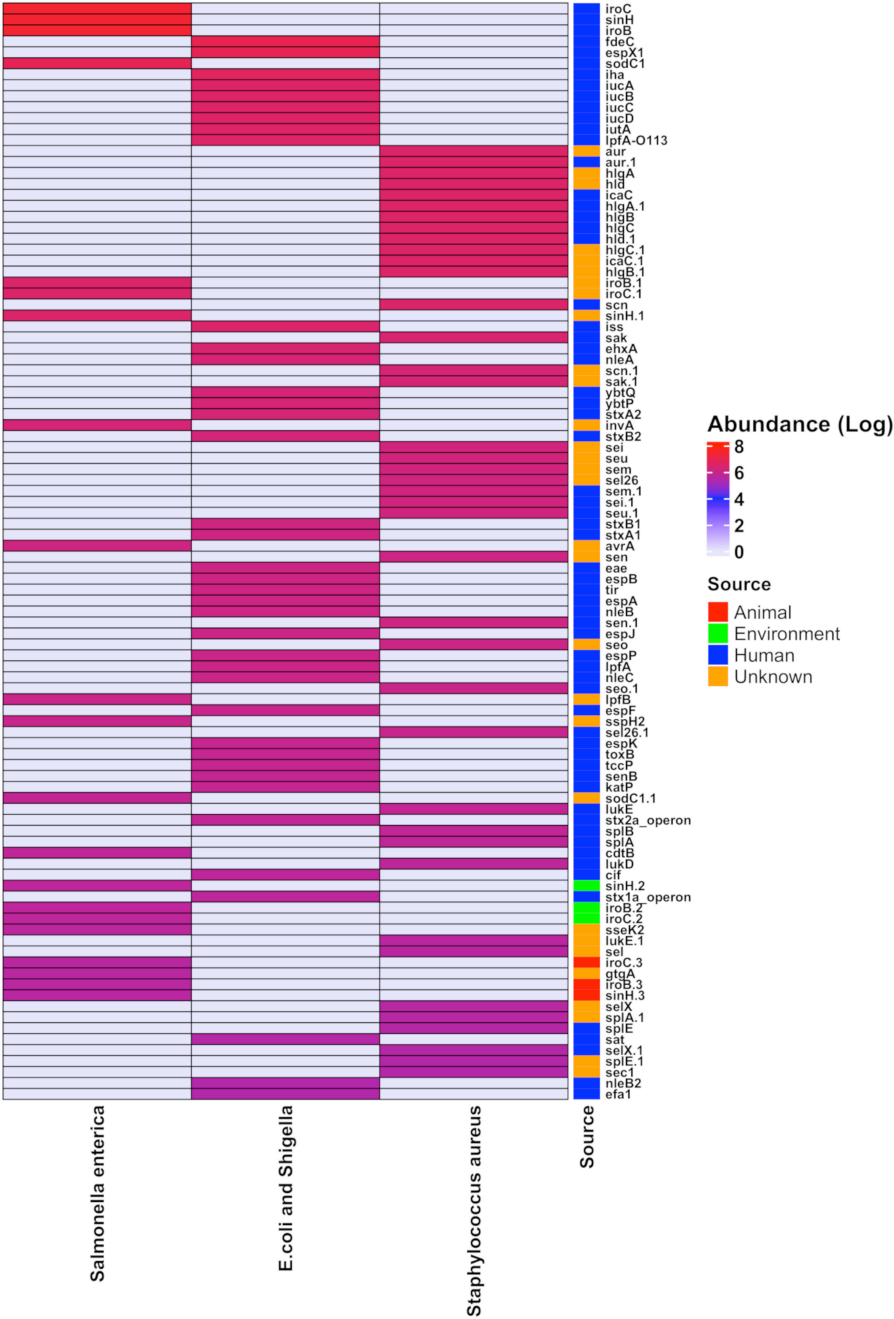
Heatmap showing the log-transformed abundance of virulence-associated genes across different bacterial pathogens. Rows represent individual virulence genes, while columns correspond to the pathogen species analyzed. Color intensity reflects relative gene abundance on a logarithmic scale, with darker red indicating higher abundance and light gray indicating lower abundance. Side annotations denote the source category associated with each gene profile, including animal, environmental, human, and unknown origins.

Two hundred and seventy-four virulence factors were identified from the pathogenic genomes. Among the 7,477 isolates identified with virulence factors, most were from human sources (*n* = 4,235, 56.6%). This was followed by isolates of unknown origin (*n* = 1,831, 24.4%), environmental sources (*n* = 896, 11.9%), and animal sources (*n* = 515, 6.8%). A total of 106 virulence factors, representing 38.7%, were shared among all four sources. Comparatively, the human source harbored 21 unique virulence factors (7.7%), while the environmental and unknown sources each exhibited 13 unique factors (4.7%). Most virulence genes were associated with a few dominant bacterial taxa. *E. coli*/S*higella* spp. accounted for the largest number of virulence genes, that is, 41.29% of all virulent factors detected, followed by *S. aureus* (15%), *S. enterica* (10%), *Bacillus cereus* (7.37%), and *Yersinia enterocolitica* (5.89%). The abundance of virulence genes is depicted in the heatmap (Fig. 10). The top five most frequently detected virulence genes are *iroC* (4%), *iroB* (4%), *sinH* (3.9%) from *S. enterica*, and *aur* (1.9%) and *hlgA* (1.9%) from *S. aureus*. In *E. coli*/S*higella* spp., 140 virulence factors were found, with *fdeC*, *espX1*, *lpfA-O113*, and *iucA* in greatest abundance. In *S. aureus*, a total of 51 virulence genes were found, with the major genes being *aur* (aureolysin), *hlgA* (gamma-hemolysin), and *hld* (delta-hemolysin). Thirty-four virulence genes were found in *S. enterica*, with the most abundant genes being *iroC* (salmochelin siderophore system), *sinH* (autotransporter adhesin protein), *iroB* (glycosyltransferase enzyme), and *sodC1* (periplasmic superoxide dismutase). Twenty-five virulence genes were found from *B. cereus*, with the major genes being *alo* (thiol-activated, cholesterol-dependent cytolysin) and *cerA* (cholesterol-dependent cytolysins). This species-gene linkage highlights that a small number of bacterial species contribute disproportionately to the overall virulence gene pool. These dominant taxa harbor multiple high-frequency virulence factors, suggesting their enhanced potential for pathogenicity and possible clinical or environmental relevance. The presence of these diverse virulent factors indicates that the isolates possess multiple mechanisms for host colonization, toxin production, and survival, contributing to their high pathogenic potential and ability to cause diverse infections.

**Fig 11:**
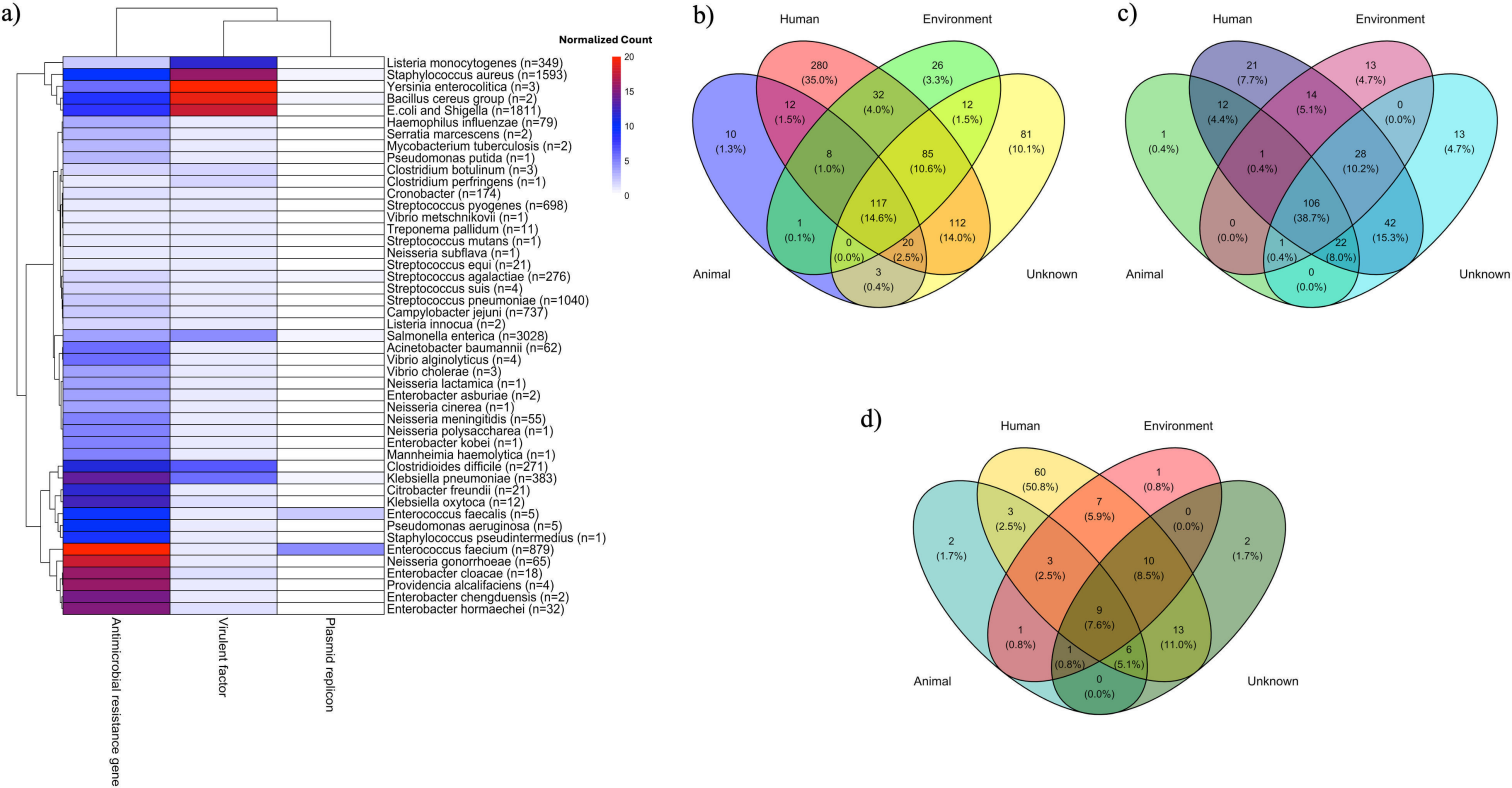
Hierarchical clustering of pathogenic isolates showing co-occurrence patterns of ARGs, virulence factors, and plasmids (**a**), based on normalized counts. Higher values are indicated in red, lower values in blue, and zero counts in white. The Venn diagrams illustrate the distribution and overlap of ARGs (**b**), virulence factors (**c**), and plasmid replicons (**d**) across different sources.

Figure 11a shows a hierarchical clustering heatmap of normalized counts of ARGs, virulence factors, and plasmid replicons across different bacterial species. Higher abundances of ARGs and virulence factors were mainly observed in clinically important pathogens such as *S. aureus*, *E. faecium*, and members of the *Enterobacteriaceae* family. The correlation analysis indicated that resistance and virulence genes frequently co-occur in several bacterial species, whereas plasmid replicons showed a more restricted distribution among taxa. The Venn diagrams show the distribution of these genomic features across human, animal, environmental, and unknown sources. ARGs were mainly detected in human-associated samples (35.6%), with 117 genes shared among all four source categories, indicating their widespread distribution (Fig. 11b). Virulence factors showed the highest level of sharing, with 38.7% present across all sources (Fig. 11c). In contrast, plasmid replicons were more source specific, with 50.8% uniquely found in human-associated samples (Fig. 11d). These results suggest that ARGs and virulence factors are widely distributed across different environments, while the mobile genetic elements involved in their transfer may be more specific to the human microbiome.

## DISCUSSION

### Risk of critical pathogens and antimicrobial resistance

The increasing co-occurrence of ARGs, plasmid replicons, and virulence determinants in pathogenic bacteria represents an escalating public health challenge. This trend is strongly influenced by the widespread use of antibiotics in human health care and animal livestock management. Our data set illustrates persistently high resistance to major antibiotic classes, particularly beta-lactams, aminoglycosides, and quinolones, consistent with post-pandemic global patterns (27, 28). The dominance of isolates originating from human sources, with fewer originating from animals and the environment, reflects existing surveillance structures that remain primarily oriented toward human health. This emphasis on clinical surveillance has facilitated the detection and monitoring of major AMR trends in Europe. For example, CAESAR surveillance data further demonstrated marked increases in key pathogens in 2021 compared with 2020, including *Acinetobacte*r spp. (50%), *K. pneumoniae* (36%), *E. coli* (26%), and *S. pneumoniae* (22%), with southern and eastern Europe experiencing the highest resistance levels (29).

In this study, we identified 799 ARGs and associated allelic variants, 274 virulence factors, and 117 plasmid replicons across clinically relevant bacterial genomes. *K. pneumoniae*, *E. coli*/*Shigella* spp., and *S. enterica* exhibited the highest burden of ARGs, consistent with their established roles in hospital and community-acquired infections. The detection of both resistance and virulence determinants on mobile genetic elements underscores the risk posed by hypervirulent, MDR pathogens. Globally, 33 major bacterial pathogens were responsible for 13.6% of all deaths and 56.2% of sepsis-related deaths in 2019, with *S. aureus*, *E. coli*, *S. pneumoniae*, and *K. pneumoniae* accounting for more than half of this burden (54.9%) (30). The WHO (2017) list of 24 priority pathogens includes many taxa also prevalent in our Irish data set, highlighting the global relevance of national resistance trends. Given the clinical importance of these priority pathogens and their occurrence across multiple reservoirs, PCA, PCoA and PERMANOVA analyses (*R*² = 0.059, *P* = 0.899) revealed shared resistance gene patterns across human, animal, and environmental sectors in Ireland, supporting the need for a comprehensive One Health surveillance framework. However, the limited data from animals and environmental sources make interpretation more difficult.

*Klebsiella pneumoniae*, a high-priority pathogen (31), showed elevated ARG abundance coupled with extensive virulence and plasmid profiles and most isolates originated from human sources. *K. pneumoniae* is responsible for approximately one-third of gram-negative nosocomial infections, including urinary tract infections, pneumonia, wound infections, endocarditis, and septicemia (32). The simultaneous detection of *fosA*, *bla_SHV-1_*, and *bla_OXA-48_*, along with *IncFIB(K)* and *IncL* plasmids and siderophore-related genes such as *ybtP* and *iucA* in this study, suggests the emergence of hypervirulent MDR lineages. Similar genomic profiles, including *iucA*, *iutA*, *bla_CTX-M-15_*, and *bla_OXA-48_*, have been reported in European isolates (33, 34). The presence of *bla_SHV-11_* and *bla_OXA-48_* is clinically significant, conferring resistance to penicillins, early cephalosporins, and carbapenems (35, 36). Despite analyzing only 383 genomes (2000–2025), *K. pneumoniae* showed substantially higher resistance levels than the other pathogens evaluated. Beta-lactams remain among the most frequently prescribed antibiotic classes in Ireland (37), and we detected 81 beta-lactam resistance genes in *K. pneumoniae* isolates, consistent with earlier findings of 156 such genes in European isolates (33). National surveillance has reported a rise in invasive *K. pneumoniae* infections, from 492 cases in 2022 to 604 in 2023, accompanied by increases in third-generation cephalosporin resistance (13.2%–15.9%) and ESBL positivity (10.8% to 12.6%) (24). This leads to evasion of host immunity, rapid dissemination, and establishment of persistent infection niches. Continued genomic characterization is therefore essential to inform treatment strategies and AMR mitigation (38). The number of *K. pneumoniae* isolates obtained from environmental (*n* = 15) and animal (*n* = 1) sources was very low, making it difficult to comprehensively discuss the One Health perspective, although *K. pneumoniae* is recognized as an important pathogen in both animals and the environment (39, 40).

*Escherichia coli*/*Shigella* isolates were characterized by high frequencies of efflux-based resistance and ARGs targeting beta-lactams, aminoglycosides, and quinolones. Predominant resistance determinants included *blaEC*, *acrF*, *mdtM*, *gyrA_S83L*, and *sul2*, reflecting diverse resistance strategies, including enzymatic degradation, efflux activity, and chromosomal target-site mutations. We identified 140 virulence genes, including *fdeC*, which facilitate epithelial adherence and biofilm formation (41), and *espX1*, which modulates host immune signaling (42). *E. coli* is ubiquitous in humans and animals, facilitating HGT and widespread dissemination of ARGs (43). Although national surveillance indicated a slight decline in MDR *E. coli* bloodstream infections from 2022 to 2023 (24), isolates deposited in the genomic database showed increasing representation of resistance determinants during 2022–2025. Ireland has some of Europe’s highest rates of STEC infections (44), and previous studies reported diverse cephalosporinase-producing *E. coli* genotypes, including numerous *bla_CTX-M_* variants, within Irish clinical settings (45). Extensive resistance in livestock-associated *E. coli* has also been documented in pigs and poultry (46, 47), and resistant isolates have been recovered from multiple environmental reservoirs including drinking water, recreational waters, wastewater, and sewage (18, 48, 49).

*Salmonella enterica*, a major cause of foodborne illness globally (50, 51), showed diverse resistance mechanisms, including efflux pumps (*mdsA* and *mdsB*) and ARGs conferring resistance to beta-lactams (*bla_TEM-1_*), aminoglycosides [*aph(6)-Id* and *aph(3″)-Ib*], sulfonamides (*sul2*), fosfomycin (*fosA7.2*), and tetracyclines [(*tet(A)*, *tet(B)*]. Plasmid replicons [*IncFII(S)*, *IncFIB(S)*, and *IncQ1*] were common, facilitating transmission of both resistance and virulence traits (52). Virulence factors such as *iroC*, *iroB*, *sinH*, and *sodC1* support iron acquisition, adhesion, and oxidative stress defense (53–55). The resistance patterns we observed align with European reports documenting multidrug-resistant *S. enterica* strains in food, livestock, and wildlife sources (56, 57). High resistance prevalence, including *tet(A/B/C)*, *aadA2*, *sul1*, *bla_TEM-1_*, and *gyrA* mutations, has been described in serovar Derby isolates from Spain and in *Salmonella* Dublin isolates from cattle in Denmark (58, 59). These findings confirm that ARGs and plasmid lineages circulate widely between human and animal reservoirs across Europe. In this study, 152 *Salmonella* serotypes were identified, with *S.* Enteritidis (*n* = 618) being the most prevalent, followed by *S. Typhimurium* (*n* = 315) and *S. Agona* (*n* = 300). *S. enterica* serotype Enteritidis is regarded as a major foodborne *Salmonella* serotype responsible for gastrointestinal illness of varying severity in humans (60).

*Staphylococcus aureus* isolates exhibited resistance to beta-lactams (*blaI*, *blaZ*, and *mecA*), tetracyclines [*tet(38)* and *mepA*], fosfomycin (*fosB*), and quinolones (*gyrA_S84L* and *parC_S80F*), alongside numerous virulence genes, including *aur*, *hlgA*, and *hld*. Previous Irish hospital studies have reported multidrug-resistant MRSA strains with substantial virulence potential (61, 62). Large-scale genomic surveys have similarly documented widespread ARG distributions, with *mecA* dominating chromosomal ARGs and *blaZ* prevalent on plasmids (63, 64). In our analysis, 153 ARGs were identified across 1,593 *S. aureus* genomes.

Additional pathogens of clinical importance (*E. faecium*, *Listeria monocytogenes*, *C. jejuni*, and *C. difficile*) also carried diverse resistance determinants. *E. faecium* isolates harbored genes conferring resistance to vancomycin (*vanS-A*/*vanY-A*/*vanA*), tetracyclines [*tet(M)*], macrolides/streptogramins [*msr(C)*], aminoglycosides [*aac(6′)-I*], and beta-lactams (*pbp5* variants), together with plasmids *repUS15*, *repUS43*, and *rep2*. No virulence genes were detected, although previous food-associated studies reported virulence factors such as *asa1* and *gelE* (65). *L. monocytogenes* genomes, though few, carried *vga(G)* and *fosX*, plasmids *rep25*/*rep26*, and virulence genes *inlC*, *lntA*, *inlK*, and *inlI*. Listeriosis remains a significant concern in Ireland, with nine cases and one probable death reported in 2025 (66). Modest increases in *L. monocytogenes* occurrences across dairy farms in the Ireland have been documented (67).

Environmental samples showed the highest contribution of non-human isolates, dominated by *S. enterica* (*n* = 291), *L. monocytogenes* (*n* = 210), *Cronobacter* (*n* = 151), and *S. aureus* (*n* = 133), along with smaller numbers of *C. jejuni*, *C. difficile*, and *A. baumannii*. Animal-derived isolates were fewer but diverse, led by *S. enterica* (*n* = 268), *E. coli*/*Shigella* spp. (*n* = 209), and *C. jejuni* (*n* = 97), with minor representation from *S. aureus*, *L. monocytogenes*, and *C. difficile*. Overall, both environments and animals act as important reservoirs for key zoonotic and foodborne pathogens, especially *Salmonella* and *Campylobacter*. Studies reported multidrug-resistant *S. enterica* and *L. monocytogenes* from environmental samples from Ireland and other EU countries (47, 68, 69).

Recent surveillance of *S. enterica*, *E. coli*, and *C. jejuni* in Irish livestock shows high resistance to commonly used antibiotics such as ampicillin and tetracycline. Multidrug resistance is frequently observed in indicator *E. coli* from pigs and poultry (70). In Ireland, tetracyclines (oxytetracycline) are widely used, particularly in pigs via medicated feed for group treatments. Sulfonamides with trimethoprim are commonly used in weaner pigs and calves. Macrolides (tylosin and tulathromycin) are frequently applied for respiratory infections in cattle and swine. Penicillins (amoxicillin and benzylpenicillin) are mainly used as injectables in calves and for intramammary therapy in dairy cows. Fluoroquinolones (enrofloxacin and marbofloxacin), classified as EMA Category B (“Restrict”), are still used on some pig farms, mainly as injectables in piglets (71).

The hierarchical clustering analysis shows a strong association between ARGs and virulence factors, especially in clinically important bacteria such as *S. aureus* and *E. coli*. The frequent co-occurrence of ARGs and virulence factors suggests that many pathogenic bacteria may acquire both resistance and virulence traits together. Although plasmid replicons were present at lower abundance, they were often found in regions with high ARG and virulence factor abundance, indicating that plasmids may help in the transfer of these genes between bacteria. The Venn diagram analyses further showed that many ARGs and virulence factors were shared across human, animal, environmental, and unknown sources, suggesting their widespread distribution. Overall, these findings indicate a positive relationship between AMR and virulence, possibly driven by mobile genetic elements involved in HGT. Several studies have reported a strong association between ARGs and virulence factors carried on plasmids, particularly in pathogens such as *E. coli* and *K. pneumoniae*. These plasmids often harbor both resistance and virulence determinants, promoting their co-selection and contributing to the rapid emergence of multidrug-resistant and highly virulent bacterial strains under antibiotic pressure (72, 73).

Although the study included a higher number of isolates from human samples, all identified species were retained in the database to provide a comprehensive overview of pathogen distribution across One Health domains. Species detected only in human, animal, environmental, or unknown sources were also included, as their absence in other domains may reflect limited surveillance or sampling rather than their true absence. Including all species helps identify existing surveillance gaps and supports future targeted investigations across different One Health sectors.

### Surveillance and policy implications

Our findings highlight the need for comprehensive AMR surveillance and comparison of ARGs across clinical, agricultural, and environmental sectors. Although Ireland participates actively in EARS-Net, these systems primarily collect phenotypic data. Our analysis demonstrates the value of WGS for identifying high-risk species and tracking major ARGs, plasmids, and virulence genes. We recommend that WGS data generated through national surveillance be made openly available and expanded across human, animal, and environmental domains to support One Health comparative analyses.

According to HPSC (66), access-group antibiotics accounted for 78% of usage in early 2025, yet overall consumption remained high at 20–21 DIDs, positioning Ireland as the ninth highest antibiotic user in Europe in 2024. Achieving the EU target of reducing consumption by 27% by 2030 (to 16.6 DIDs) will require sustained stewardship interventions (66). While Ireland’s Second National Action Plan on Antimicrobial Resistance (2021–2025) has strengthened awareness, surveillance, infection prevention, and responsible prescribing, our data suggest additional targeted strategies are needed, particularly for high-risk clinical and agri-food-associated pathogens (74).

### Limitations

This study has several limitations. First, the NCBI Pathogen Detection data set is unevenly represented across species and sources, which may introduce sampling bias. Second, the analysis is entirely genomic and lacks laboratory-based phenotypic validation. As a result, associations between plasmids, ARGs, and virulence factors are predictive and require experimental confirmation.

### Conclusion

Our results indicate a persistent and evolving antimicrobial resistance burden in Irish pathogenic bacteria, characterized by the notable co-occurrence of resistance, virulence, and plasmid genes with clinical relevance. These findings highlight the urgent need to strengthen genomic surveillance, use antibiotics more responsibly, and strengthen One Health efforts across humans, animals, and the environment. While our *in silico* approach offers rapidly gained insights into resistance patterns, follow-up studies require phenotypic validation to establish clinical significance and inform targeted interventions. The current study offers baseline data to inform policymakers, enabling timely interventions and the implementation of control measures in identified critical pathogens. We recommend adopting alternative therapeutic strategies to mitigate the overuse of antibiotics and support the long-term preservation of their efficacy.
